# Strengthening funding and integration for NTD elimination: insights from the 2025 Annual Meeting of National NTD Programme Managers in the WHO African Region

**DOI:** 10.1186/s12919-025-00357-x

**Published:** 2026-01-26

**Authors:** Elizabeth Juma, Dorothy Achu, Abbie Barry, Abate Beshah, Ako A. G. Adjami, Amir B. Kello, Augustin K. Ebeja, Balla Jatta, Didier Bakajika, Dyesse Y. Nduba, Dyuti Sen, Flora M. Lekhanya-Mohatla, Honorat G. M. Zoure, Jorge Cano, Koku M. Davi, Moussa S. Sanfo, Namuchile Kaonga, Pauline Mwinzi, Santa-Mika Ndayiziga, Yves T. M. Barogui, Yuka Makino, Bridget Farham, Benido Impouma

**Affiliations:** 1 World Health Organization Regional Office for Africa, Cité de Djoué, B.P. 06, Brazzaville, Republic of Congo; 2 NTD Programme, Ministry of Health, Banjul, The Gambia; 3 WHO Country Office, Lomé, Togo

**Keywords:** Neglected tropical diseases (NTDs), Health systems integration, Programme sustainability, Digital health, Artificial intelligence, Africa

## Abstract

The 2025 Annual Meeting of National Neglected Tropical Disease (NTD) Programme Managers (2025 PMM) in the WHO African Region convened stakeholders in Lomé, Togo, under the theme “Innovating for Acceleration: Pathway to NTD Elimination.” A key focus was the changing funding environment and the necessity for enhanced integration of NTD services within health systems to guarantee sustainable advancement toward the 2030 elimination goals. The conference was convened in the context of substantial disruptions stemming from the USAID funding pause, which interrupted essential mass drug administration (MDA) programmes and epidemiological monitoring activities across multiple nations. Country experiences highlighted the fragility of external funding dependence and underscored the importance of domestic resource mobilization, decentralized implementation, and programmatic integration. Strategic discussions highlighted opportunities to incorporate NTD services into national health financing mechanisms, and routine health campaigns, alongside leveraging digital tools and partnerships. Participants emphasized the urgency of political commitment, sustained investments, and integrated service delivery models to build resilience and close equity gaps. The meeting further underscored the need for bold, country-led responses and multisectoral collaboration to advance NTD elimination efforts in a rapidly evolving global health financing environment.

## Introduction

Neglected tropical diseases (NTDs) comprise at least 21 largely preventable and treatable conditions listed by WHO, that disproportionately affect the poorest communities in tropical and sub-tropical regions [1, 2]. In 2012, the World Health Organization (WHO) released its first NTD Road Map; the current 2021–2030 edition calls for eliminating at least one NTD in 100 countries, reducing the number of people needing treatment for NTDs by 90%, reducing disability-adjusted life years related to NTDs by 75% and eradicating two NTDs by 2030 [3–5].

The WHO African Region bears roughly 40% of the global NTD burden and is endemic for 20 of the NTDs except for Chagas disease: all 47 member states are endemic for at least one NTD, and 37 have five or more NTDs [4, 6]. Progress is tangible—42 countries are certified Guinea-worm-free, nine have eliminated trachoma as a public-health problem, and Togo and Malawi have eliminated lymphatic filariasis [7–10]. Togo is the first country in the world to have eliminated 4 NTDs. Yet persistent funding gaps, limited health-system integration, and other systemic vulnerabilities threaten attainment of the 2030 targets [11–13].

**Fig. 1:**
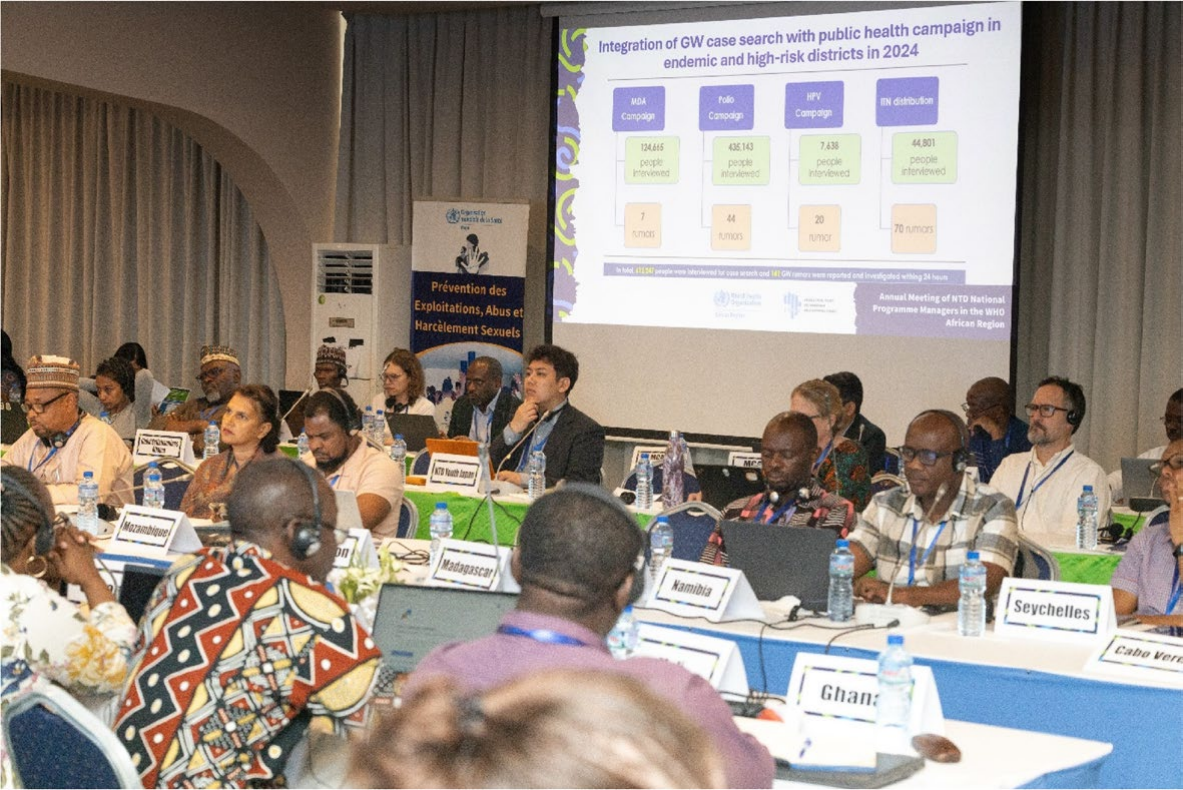
Participants at the 2025 Annual Meeting of National NTD Programme Managers in the WHO African Region

WHO convenes stakeholders, issues guidance, and helps countries develop strategic frameworks, such as the NTD Master Plan Framework for development 2021–2025 (https://espen.afro.who.int/tools-resources/documents), or more disease-specific guidance such as the visceral leishmaniasis elimination initiative in eastern Africa [14], and assists in the preparation and submission of dossiers for validation, verification and certification of elimination [15]. Its annual Programme Managers’ Meetings (PMMs), held since 2016, provide a platform to review progress, share lessons, and align regional priorities. The 2025 PMM, held in Lomé, Togo from 15–17 April 2025 under the theme *“Innovating for Acceleration: Pathway to NTD Elimination,”* marked the roadmap’s midpoint and focused on integrating NTD services into national systems amid shrinking external finance. Co-organized by the WHO Regional Office for Africa and the WHO country office in Togo, the meeting was attended by national programme managers, implementing partners, donors, and WHO staff, serving as a key forum for strategic dialogue on integrating NTD services into national health systems and mitigating the impact of declining external financing (Fig. 1).

In this paper we synthesize the key insights from the plenary sessions of the 2025 Annual Meeting of National NTD Programme Managers in the WHO African Region. We focus on the core themes discussed collectively by all participants including the evolving funding landscape, integration of NTD services within health systems, the role of innovative surveillance approaches, and the application of artificial intelligence in enhancing programme impact. Drawing from country experiences and partner contributions, the paper captures practical innovations, lessons learned, and actionable recommendations aimed at sustaining programme gains, promoting country ownership, and accelerating progress toward the 2030 NTD elimination targets in the African Region.

### Challenges and opportunities for NTD elimination

#### Mid-term milestones and integration gaps

Tables 1 and 2 summarize the progress achieved, key challenges encountered, and priority actions identified by facilitators regarding case management NTDs (CM-NTDs) and preventive chemotherapy NTDs (PC-NTDs), respectively.

**Table 1:** Progress, Challenges, and Priority Actions for CM-NTDs in the WHO African Region

NTD	Progress	Challenges	Priority actions/Recommendations
Guinea Worm	• 42 of the 47 countries in the African region certified so far • 5 countries remain endemic: Angola, Cameroon, Chad, Ethiopia and South Sudan • Only 15 human cases reported in 2024 (9 in TCD, 6 in SSD)	• Insecurity • Animal infection • Lack of funding	• National ownership • Advocacy and awareness • Multi-sectoral collaboration, OneHealth approach • Enhanced surveillance
Yaws	• 12 countries, never considered as endemic are suspected free of yaws, but are yet to be certified • Since 2020, three countries (Cameroon, Central African Republic, Congo) are implementing Total Community Treatment (TCT) with azithromycin for Yaws eradication • Integrated surveillance and total target treatment (TTT) ongoing • Regional consultation for yaws eradication in Africa	• Lack of funding: Implementation & access to tests (RDT, DPP Syphilis Screen & Confirm Assay) • Weak integrated surveillance for yaws in countries • Unknown yaws status in 26 countries	• Certification for no history countries: Questionnaire shared with countries • National ownership • Advocacy for resources mobilization • Enhanced integrated surveillance
Human African Trypanosomiasis (HAT)	• 9 Countries validated for Elimination of HAT as PHP	• Inadequate funding • As cases decline, maintaining robust passive and reactive surveillance becomes more difficult and expensive • Existing tests require specialized equipment or training, and field-friendly, highly sensitive tools are still under development or not widely available	• Expand targeted active case finding in residual hotspots and hard-to-reach populations • Strengthen laboratory diagnostic capacity by ensuring availability of screening tools (e.g. RDTs, microscopy, LAMP, mAECT) at peripheral levels • Advocacy and domestic funding
Leprosy	• 46 countries achieved and sustained Leprosy elimination as PHP • 8 countries close to achieve interruption of transmission (*Algeria, Botswana, Eritrea, Eswatini, Lesotho, Mauritius, São Tomé and Príncipe and Seychelles)*	• Lack of funding & weak national ownership • Dwindling expertise • Late detection o G2D rates: 2.7 million per Pop o Ongoing transmission (2.6 million per Child Pop.) • Highly endemic pockets/Countries • Insecurity and Periodic outbreaks	• Enhance surveillance: contact tracing & PEP, integrated with active case detection • Continue piloting Leprosy elimination monitoring tools (LEMT) • Enhance integrated capacity building • Strengthen advocacy, partnerships to enhance political commitment & resources mobilization
Visceral leishmaniasis (VL)	• Number of cases reported decreased by 62% (from 11,119 in 2014 to 4,323 in 2023) • The African region accounts for 37% of the global burden • Reduction in case fatality rates in countries • Access to services improved, not adequate • Countries adapted national guidelines for VL. No. reporting countries improved • Framework for VL elimination launched, June 2024 (MoU signing at 78th WHA)	• Lack of funding and weak national ownership • Climate change (new foci, out breaks, etc.) • Imperfect tools • Insecurity and displacement	• Signing of MoU for the implementation of the VLE Framework (side event at 78th WHA) • Guideline adaptation by national programmes • Advocacy and resource mobilization including domestic funding • Mapping, surveillance, cross border collaboration, active case finding • Research & Development
Cutaneous leishmaniasis (CL)	• Implementation of integrated skin NTD approach • 15 out of the 19 endemic countries in the region reporting • Number of countries reporting increased • Annual incidence: 11,777 (65% Algeria) in 2023 • 30% reduction in incidence between 2017 and 2023 • National programme developed & implementing CL guidelines • Capacity of HWs in countries improved	• Lack of resource • Inadequate knowledge on disease burden and distribution • Lack of tools: non-invasive and rapid tool for diagnosis • Treatment is often expensive, invasive and toxic	• Promote implementation of integrated skin NTD approach • Advocacy and resource mobilization • Burden assessment and reporting • Research & Development for diagnostic, treatment and prevention tools
Buruli ulcer	• Reduction of over 70%: 5,871 in 2004 ↘ 1,573 in 2023 • Approximately 30% of reported cases over the past five years were classified as Category III lesions, but still significantly above the target of less than 10% • Proportion of laboratory-confirmed cases increased from 10% in 2019 to 31% in 2023, although still below the 95% target set for 2030 • Treatment completion for Buruli ulcer improved from 70% in 2019 to 80% in 2023, though still below the target of over 98%	• Lack of funding and weak national ownership • Exact mode of transmission is still unknown • Lack of point-of-care diagnostic test • Weak surveillance system in some countries • Long duration of the treatment (8 weeks)	• Strengthen integrated surveillance • Development of new medicines with the potential of reducing the duration of the treatment • Enhance advocacy and resource mobilization • Development of RDT to ensure early diagnosis
Rabies	• Ongoing collaborative works to scale up critical actions for rabies elimination in Member States	• Discrepancies in data reported for rabies • Lack of reliable data/sub optimal surveillance	• Scaling up integrated surveillance, diagnostic, reporting & response • Advocate for post-exposure use of human vaccines • Support countries for Gavi rabies vaccines application • Regional stakeholders' meetings • Integrated Bite Case Management (IBCM)
Noma	• Development of a disease summary for noma in line with those included in the NTD road map 2021 − 2030, including targets (under development)	• Aetiology & pathogenesis unknown • Diagnosis is purely clinical • High fatality rate with severe disability, stigma, and discrimination • Limited awareness and knowledge, and inadequate resources • Poor multisectoral coordination and stakeholder engagement • Weak surveillance systems and a lack of research	• Inclusion of noma in national NTD MPs • Integration of noma into surveillance systems and active case-detection in high-endemic areas • Partnerships and communities of practice • Communication and advocacy strategy • Capacity building to prevent, detect, treat • Resource mobilization • Research prioritization

**Table 2:** Progress, Challenges, and Priority Actions for PC-NTDs in the WHO African Region

NTD	Progress	Challenges	Priority actions
Trachoma	• 6 countries validated to have eliminated trachoma as a public health problem (Ghana, Gambia, Togo, Malawi, Benin, & Mali)	• Pending completion of mapping surveys) • Delineation of Oncho Transmission zones (OTZs) • Pending completion of Impact assessment ✓ Reaching the TT threshold for EPHP ✓ Persistent and recrudescent districts/hot spots ✓ Hard to reach areas & special populations ✓ Cross-border issues ✓ Insecurity ✓ Lack of funding	• Supporting completion of mapping surveys • Supporting delineation of OTZ • Supporting completion of Impact assessment • Reaching 100% geographic coverage for MDA and TT surgery • Addressing persistent and recrudescent districts • Reaching special populations & “insecure” areas • Cross-border collaborations • Supporting countries with elimination dossiers • Post-validation surveillance • Resource mobilization
Soil Transmitted Helminths	• 13 countries have achieved ≤ 2% moderate/heavy intensity prevalence pending validation of elimination of STH as a public health problem		
Schistosomiasis	• Dossier preparation for validation of elimination of Schistosomiasis as a public health problem (< 1% heavy intensity prevalence) currently on-going in Algeria		
Lymphatic Filariasis	• Eliminated as a public health problem in two countries (Togo and Malawi) • 20 countries implementing post MDA or post validation surveillance, up from 14 in 2020		
Onchocerciasis	• Niger became the first country to be certified free of onchocerciasis • 7 countries stopped MDA for ≥ 1 focus		

While substantial progress has been made—as of the time of submitting this manuscript, at least one neglected tropical disease (NTD) has been eliminated in over 57 countries globally—significant challenges remain in achieving the 2030 NTD elimination targets [16]. Within the WHO African Region, presenters highlighted several major milestones: 42 of 47 countries have been certified free of Guinea worm disease [17, 18], and nine countries have achieved validation for the elimination of trachoma as a public health problem [8], including Burundi and Senegal in July 2025. Togo [7] and Malawi [10] have eliminated lymphatic filariasis (LF) as a public health problem, and Niger became the first country to be verified for the elimination of onchocerciasis in the African region. Algeria is in an advanced stage of preparing its dossier for validation of the elimination of schistosomiasis as a public health problem, and this process has also been initiated in Mauritius. In parallel, several countries — including Niger, Mali, Burkina Faso, and Namibia — are believed to be ready to reach a similar milestone for soil-transmitted helminths (STH) and are awaiting the validation process. Progress has also been reported for case management NTDs, including a 62% reduction in visceral leishmaniasis cases between 2014 and 2023, and an over 70% reduction in Buruli ulcer cases since 2004. Nine countries have been validated for the elimination of human African trypanosomiasis (HAT) as a public health problem, and 46 countries have achieved and sustained leprosy elimination as public health problem, with eight nearing interruption of transmission.

Despite these gains, presentations underscored persistent barriers impeding further progress. These include incomplete disease mapping, fragile surveillance systems, and limited integration of NTD services into routine health delivery. while 42 of the 47 countries in the African region have now been certified Guinea worm disease (GWD) free, transmission continues in five countries, mainly due to insecurity and animal infection. Similarly, although 13 countries have reached ≤ 2% moderate/heavy intensity prevalence for STH—a key threshold for validation—many others continue to face challenges in sustaining MDA coverage and access to WASH services[19–21].

Integration efforts remain uneven across the region, with most NTD programmes still operating in vertical silos and heavily reliant on donor-led campaigns [22, 23]. This fragmentation hinders the efficient use of health system resources and limits the sustainability of interventions. Participants emphasized the urgent need for more coordinated and systematic incorporation of NTD interventions into national health strategies, particularly within primary health care and Universal Health Coverage (UHC) frameworks. Strengthening national ownership, aligning disease-specific efforts under common monitoring and planning platforms, and improving coordination across sectors were identified as critical steps toward resilient and country-owned NTD programmes.

#### Current funding landscape for NTDs (311 words)

**Fig. 2:**
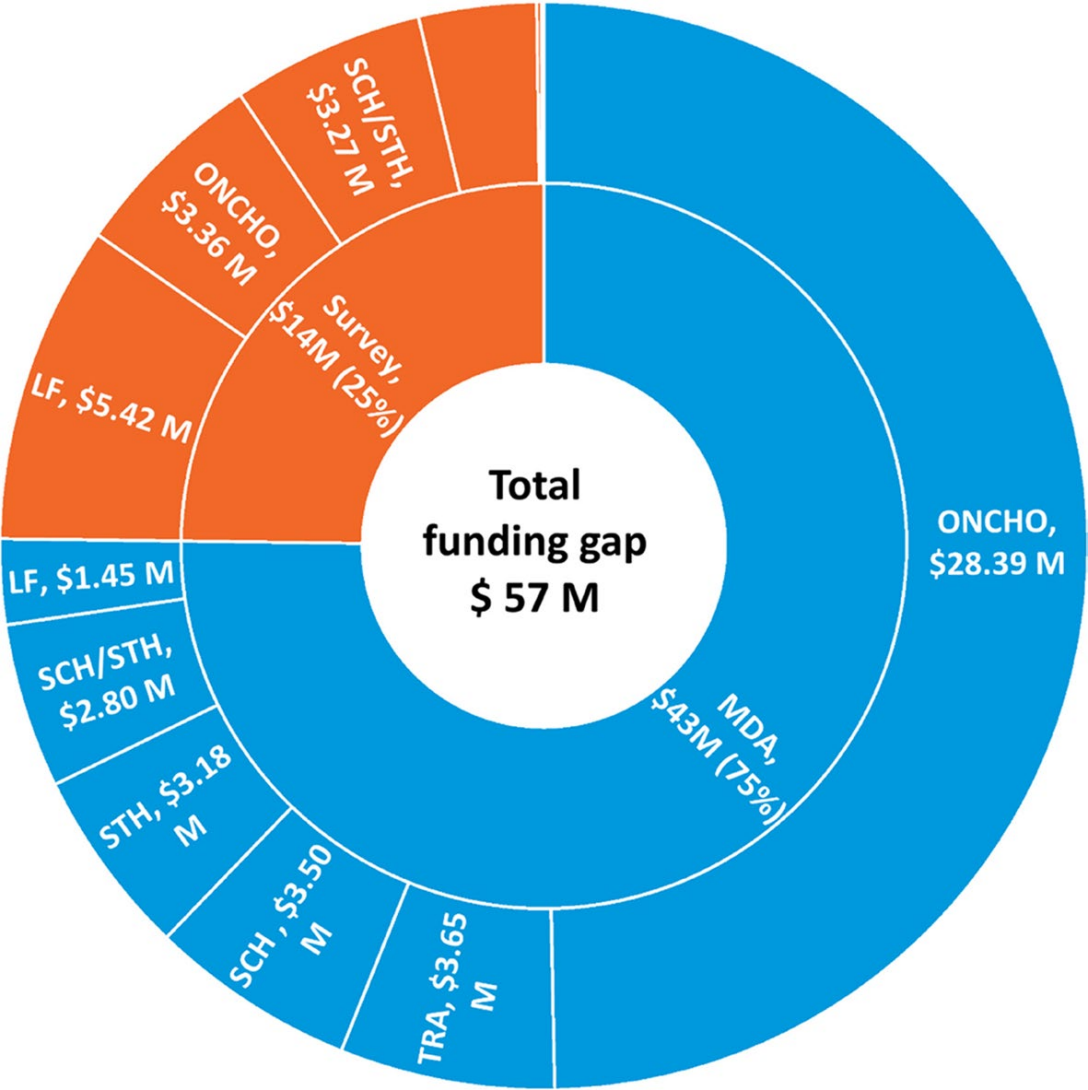
Estimated funding gap by activity and disease, WHO African Region, 2025

The sudden halt of three major USAID projects —including *Act to End NTDs I East*, *Act to End NTDs I West*, and *Ending Neglected Diseases through Operational Research (ENDOR)*— in early 2025 disrupted NTD activities in 24 African countries. More than 114 planned actions—including 47 mass drug administration (MDA) campaigns meant for ≈142 million people—were suspended, leaving an estimated US $57 million gap, three-quarters of it for MDAs and the remainder for critical surveys (Fig. 2).

This crisis has exposed the vulnerability of NTD programmes heavily reliant on external financing [24] and underscored the need for strategic transition toward domestic resource mobilization and financial sustainability [25]. Presentations from WHO and regional institutions outlined a global mitigation framework prioritizing high-risk MDAs, expiring drug inventories, and essential surveys linked to elimination milestones. Countries were encouraged to leverage integration with other health campaigns [26] and strengthen the use of planning tools such as the Joint Application Package (JAP) and the Expanded Special Project for the Elimination of Neglected Tropical Diseases (ESPEN) IU Planner (https://espen.iuplanner.app/) to track resource needs and support advocacy efforts.

Country responses illustrate different pathways to resilience. Benin decentralised quickly, financing MDAs through local budgets and community systems. Ethiopia reprioritised activities to partially address the gaps, redistributed medicines, and pushed to include NTD commodities in national procurement. Angola drafted a transitional financing plan built on multisectoral partnerships, while Mainland Tanzania embedded NTD budgets in district plans and national supply chains.

Across cases, four lessons stand out: (i) high-level political backing, (ii) flexible and decentralised programme design, (iii) integration of NTD services into primary health care and medicine logistics platforms, and (iv) diversification of funding—from domestic budgets to public–private partnerships and health insurance schemes. Implementing these measures, as encouraged by the WHO Sustainability Framework [4], is essential for shielding NTD programmes from future funding shocks and sustaining progress toward the 2030 goals.

### Innovation and technology for improved NTD management

#### Innovative integrated surveillance tools: enhancing data for NTD elimination

Timely, high-quality, and geographically precise data are now indispensable for achieving the 2030 NTD targets [27]. Plenary discussion highlighted three complementary digital innovations that together reshape how programmes collect, analyse, and act on field information.

The ESPEN Collect platform, developed and managed by ESPEN, is a digital data collection system designed to support standardized, field-based data gathering for NTD programmes across the African region. Originally established to streamline data capture for epidemiological surveys targeting preventive chemotherapy NTDs, ESPEN Collect has evolved into a versatile tool that facilitates real-time, georeferenced data collection using mobile devices, even in offline settings. More information on the platform is available at: https://espen.afro.who.int/tools-resources/data-collection-tools/espen-collect.

Recent enhancements to the ESPEN Collect platform have significantly improved its functionality for thorough onchocerciasis vector surveillance. New modules have digitized essential aspects of the surveillance process, including river prospection, blackfly collection, and PCR-based laboratory validation. Furthermore, the platform features real-time dashboards and geospatial machine learning models that enhance the identification of potential vector breeding sites, thus optimizing operational efficiency and enabling focused vector surveillance. These enhancements were initially piloted in Nigeria and are currently being deployed to other countries within the WHO African Region.

The ESPEN Geospatial Microplanner—developed under WHO’s ESPEN initiative—helps African NTD programmes translate high-resolution geospatial data into practical micro-plans [28]. By combining travel-time modelling with openly available layers on population, health-facility location, and road networks, the tool lets managers define catchment areas down to sub-district level, pinpoint underserved communities, and refine target-population estimates. It couples this technical depth with a familiar Excel workflow, automating aggregation, progress tracking, and one-click export to national systems such as DHIS2. Requiring minimal training, the ESPEN Microplanner tool is already being used by national and sub-national teams to sharpen campaign planning, allocate resources more equitably, and align NTD activities with broader health-service delivery. (See tool overview: https://espen.afro.who.int/tools-resources/advanced-analytical-tools/espen-geospatial-microplanner).

The Schistosomiasis Practical and Precision Assessment (SPPA) tool supports sub-district treatment decisions through a two-stage approach that pairs broad mapping with targeted sampling in epidemiologically uncertain zones [29, 30]. The application of SPPA in Kenya [29] revealed undetected high-burden areas at the sub-county level, enabling more targeted mass drug administration and improved resource allocation. In Senegal, the tool facilitated a shift from generalized treatment to focused intervention, reducing overtreatment in low-risk areas and improving the efficiency of the programmes. The integration of SPPA with platforms such as ESPEN Collect and Metabase© allowed for real-time data visualization and evidence-based decision-making.

More broadly, such digital innovations are transforming NTD surveillance by generating granular data, integrating with national information systems, and enabling responsive planning [31]. Meeting participants agreed that sustained investment, local capacity-building, and technical support are now critical to embed these tools in routine practice and, ultimately, to accelerate progress toward the 2030 elimination targets [32, 33].

#### Harnessing AI & data analytics: transforming NTD management for impact

A key highlight of the 2025 Annual Meeting was the exploration of how artificial intelligence (AI) and advanced data analytics can revolutionize the management of NTD [12]. With the increasing availability of digital tools, national programmes are leveraging technology to improve disease detection, strengthen surveillance, enhance microplanning, and support timely and data-driven decisions.

A new WHO mobile application, built on deep-learning image analysis, allows peripheral health workers to photograph suspected skin lesions and receive an on-the-spot, ranked list of likely NTD diagnoses. Pilots in Kenya reported > 75% sensitivity for priority conditions such as yaws and scabies, while automatic geo-tagging feeds cases into DHIS2 dashboards for immediate follow-up and more refined mapping of transmission foci [34, 35].

The Ministry of Health in Ghana introduced an innovative approach that leverages a ChatGPT-powered virtual assistant, named "NTD Assist," to support community drug distributors during mass drug administration campaigns. This virtual assistant, deployed through the WhatsApp platform, provides real-time guidance on eligibility criteria, drug administration protocols, adverse event management, and reporting procedures. Complemented by an electronic data capture tool for Android devices, this technological solution has reduced the reliance on supervisory personnel, enhanced treatment coverage monitoring, and empowered frontline health workers with immediate access to accurate information.

Additionally, the ESPEN GenAI Assistant, developed by the WHO Regional Office for Africa and partners, was showcased as a generative AI tool designed to streamline access to NTD datasets and technical resources housed on the ESPEN portal (https://espen.afro.who.int/). The assistant enables programme managers to query data tables, technical documents, and planning tools using natural language, significantly reducing the time spent searching for relevant materials and supporting more informed decision-making.

Delegates agreed that AI tools can speed diagnosis, sharpen micro-planning and empower frontline staff [36], but stressed three prerequisites for scale-up: reliable digital infrastructure, clear data-governance safeguards and targeted workforce training [37]. Integrating these tools with existing national information systems—rather than running them in parallel—will be essential to secure sustainability, country ownership and, ultimately, faster progress toward the 2030 elimination goals.

#### Strengthening NTD elimination in the WHO african region through innovation, integration, and investment

The speakers at this session stressed that achieving the 2030 NTD Road Map targets hinges on moving from donor-driven, vertically organised programmes to country-led models that are integrated and sustainably financed [22, 38]. Innovation, they noted, must encompass not only technology but also programme design, workforce incentives, financing instruments, and multisectoral partnerships [39]. Country examples (Table 3) showed that integrated campaign delivery can improve coverage, build health-system resilience, and advance universal-health-coverage goals while making better use of limited resources.

**Table 3:** Country Case Examples of Integration Strategies for NTD Programme Implementation in the WHO African Region

Country	Integration strategy	Comment
**Ethiopia**	Integrating Guinea worm surveillance into routine immunization and nutrition campaigns	Significantly reduced operational costs
	Active noma case-finding surveillance integrated with Onchocerciasis MDA	Three undiagnosed early-stage noma cases were identified and treated
**Ghana**	Integrated leprosy contact tracing and chemoprophylaxis within national health information systems and established robust monitoring frameworks. Ghana also integrated tuberculosis screening and dermatological assessments within campaign activities	Over 14,000 leprosy contacts were screened, and 11,800 received prophylaxis with single dose of rifampicin
**Madagascar**	Combined lymphatic filariasis MDA with polio vaccination	Achieved coverage above 80% while saving over $1 million in implementation costs
**Senegal**	Integrate preventive chemotherapy for malaria (seasonal malaria chemoprevention) and schistosomiasis and STH (Praziquantel and Albendazole MDA)	Feasibility and high acceptability of integrated delivery Integration was cost-effective, leveraging joint planning and logistics to reduce operational costs

A standout example of non-technological innovation was the roll-out of mobile-money payments to frontline campaign workers. Building on lessons from polio eradication, WHO and national partners created a three-phase digital-payment model—*Know Your Customer* (KYC) verification process, real-time attendance tracking, and automated post-campaign disbursement—piloted in several countries [40, 41]. Early results show faster, more transparent payments, lower transaction costs, and leading to higher worker morale [42]. An interoperable platform is now under development to serve multiple disease programmes region wide.

These experiences collectively reinforce a central theme of the meeting: integration is not simply a delivery strategy—it is a sustainability imperative. Institutionalizing NTD services within national health systems, including through primary health care platforms [22], essential health benefit packages, and national health insurance schemes, was strongly encouraged. Similarly, integrating NTD commodity supply chains into national logistics systems and aligning surveillance frameworks with broader health monitoring mechanisms were identified as critical enablers of system resilience [43].

Finally, the meeting underscored the urgency of diversifying finance. With external aid under pressure, countries were urged to expand domestic budget lines [44], tap public–private partnerships, explore debt-for-health swaps, and quantify economic returns from NTD investment. The African Union Commission called for enhanced accountability in domestic financing, urging member states to monitor progress toward the Abuja Declaration commitment. Regional financing instruments—including those from the African Development Bank, IDA21, and climate-health adaptation funds—were recommended as complementary avenues to bridge remaining investment gaps. Stakeholders agreed that sustained elimination will require steadfast domestic leadership, catalytic investment, and the mainstreaming of integrated, digitally enabled strategies capable of reaching all affected communities.

## Conclusions

The 2025 Annual Meeting of National NTD Programme Managers offered a critical mid-term reflection on the progress, challenges, and strategic imperatives for advancing toward the 2030 elimination targets in the WHO African Region. The discussions underscored that while notable milestones have been achieved—including country-level eliminations, advances in surveillance, and technological innovation—several persistent systemic challenges continue to impede sustainable progress. Paramount among these challenges is the substantial dependence on external financial support, the fragmented nature of service delivery, and the constrained incorporation of NTD interventions into national healthcare infrastructures. Advocacy emerges as a pivotal mechanism for securing funding and ensuring the enduring sustainability of public health initiatives, especially in resource-limited settings where health budgets face considerable constraints [45].

The abrupt cessation of major USAID-funded NTD projects in early 2025 starkly revealed the fragility of donor-dependent programme models, underscoring the urgent need for more resilient and sustainable approaches. Country case studies demonstrated that programmes with decentralized structures, domestic financing mechanisms, and integrated service delivery platforms were better equipped to absorb external shocks and sustain essential interventions. This shift reflects a broader transformation in the landscape of NTD control, characterized by a growing emphasis on domestic resource mobilization and programmatic integration. While international collaborations and philanthropic investments have driven substantial progress, the long-term sustainability of NTD programmes will depend on their full integration into national health systems and the establishment of reliable domestic funding streams, particularly through alignment with primary health care and UHC frameworks [22, 38].

At the same time, the meeting highlighted the transformative potential of innovation—both technological and programmatic—in advancing NTD elimination efforts. Recent advances in digital surveillance, data analytics, and artificial intelligence are enabling more precise targeting, strengthening decision-making, and improving overall programme efficiency [13, 27, 31]. Equally transformative are programmatic innovations such as mobile-money payments, which have improved worker motivation and transparency. When coupled with interoperable data systems and campaign management tools, these innovations contribute to building more accountable and responsive delivery systems [40].

In conclusion, the 2025 Annual Meeting re-affirmed that achieving the 2030 NTD targets in the WHO African Region will require a sustained shift toward country ownership, strategic integration, and innovation-driven delivery. Success will depend on how effectively countries can institutionalize NTD services within health systems, leverage digital and AI-enabled tools for programme efficiency, and mobilize domestic and regional financing. The pathway to NTD elimination is no longer defined solely by disease control metrics—it is intrinsically linked to strengthening health systems, enhancing equity, and reaffirming health as a public good. As countries move into the second half of the NTD Roadmap period, these guiding principles must anchor both national strategies and global partnerships.

## Data Availability

All presentations and materials from the 2025 NTD Programme Managers meeting in the WHO African Region are available for download at the following link: https://espen.afro.who.int/updates-events/events/6th-annual-meeting-national-ntd-programme-managers

## Abbreviations

AI: Artificial Intelligence;
AU: African Union
CM-NTDs: Case Management Neglected Tropical Diseases
DHIS2: District Health Information System 2
ESPEN: Expanded Special Project for the Elimination of Neglected Tropical Diseases
GWD: Guinea Worm Disease
HAT: Human African Trypanosomiasis
IU: Implementation Unit
JAP: Joint Application Package
KYC: Know Your Customer
LF: Lymphatic Filariasis
MDA: Mass Drug Administration
NTDs: Neglected Tropical Diseases
PC-NTDs: Preventive Chemotherapy Neglected Tropical Diseases
PHP: Public Health Problem
PMM: Programme Managers Meeting
RDT: Rapid Diagnostic Test
SPPA: Schistosomiasis Practical and Precision Assessment
STH: Soil-Transmitted Helminths
UHC: Universal Health Coverage
VL: Visceral Leishmaniasis
WHO: World Health Organization
